# Health Insurance Coverage by Occupation Among Adults Aged 18–64 Years — 17 States, 2013–2014

**DOI:** 10.15585/mmwr.mm6721a1

**Published:** 2018-06-01

**Authors:** Winifred L. Boal, Jia Li, Aaron Sussell

**Affiliations:** ^1^Division of Surveillance, Hazard Evaluations and Field Studies, National Institute for Occupational Safety and Health, CDC, ^2^Spokane Mining Research Division, National Institute for Occupational Safety and Health, CDC.

Lack of health insurance has been associated with poorer health status and with difficulties accessing preventive health services and obtaining medical care, especially for chronic diseases (*1*–*3*). Among workers, the prevalence of chronic conditions, risk behaviors, and having health insurance has been shown to vary by occupation (*4*,*5*). CDC used data from the 2013 and 2014 Behavioral Risk Factor Surveillance System (BRFSS) to estimate the prevalence of having no health care coverage (e.g., health insurance, prepaid plans such as health maintenance organizations, government plans such as Medicare, or Indian Health Service) by occupation. Among all workers aged 18–64 years, the prevalence of being uninsured declined significantly (21%) from 16.0% in 2013 to 12.7% in 2014. In both years there were large differences in the prevalence of being uninsured among occupational groups, ranging from 3.6% among the architecture and engineering occupations to 37.9% among the farming, fishing, and forestry occupations in 2013 and 2.7% among community and social services; and education, training, and library occupations to 37.0% among building and grounds cleaning and maintenance occupations in 2014 (p<0.001). In 2014, more than 25% of workers in four occupational groups reported having no health insurance (construction and extraction [29.1%]; farming, fishing, and forestry [34.6%]; food preparation and serving related [35.5%]; and building and grounds cleaning and maintenance [37.0%]). Identifying factors affecting differences in coverage by occupation might help to address health disparities among occupational groups.

BRFSS is an annual, state-based, random-digit–dialed landline and cell phone survey of noninstitutionalized adults aged ≥18 years residing in the United States.* Industry and occupation was first available as an optional module in BRFSS in 2013. In both 2013 and 2014, 17 states^†^ asked all survey participants about their health care coverage^§^ and asked participants who were currently or recently employed at the time of their interview about their industry and occupation.^¶^ Participants’ responses were coded to the 2002 version of U.S. Census Bureau occupation numeric codes. Census occupation codes were then grouped for analysis into major groups using the 2000 Standard Occupational Classification System. During 2014, 12 of the 17 states elected to expand Medicaid eligibility to persons with an income ≤138% of the federal poverty level,** and five did not (*6*).

The subpopulation of interest included respondents aged 18–64 years and currently employed for wages or self-employed in the 17 states. It excluded those on active military duty or whose occupation was missing or could not be coded. Respondents aged ≥65 years were excluded because they were presumed to be eligible for Medicare (*7*).

Data were weighted and analyzed to account for the complex BRFSS sampling design. The prevalence of being uninsured was estimated by occupational group and sociodemographic characteristics, stratified by year. Unadjusted prevalences by occupational group were calculated to present the magnitude of noncoverage for each occupational group. To control for effects of the potential confounders age, sex, race/ethnicity, language in which the survey was conducted, education, annual household income, marital status, employment status (currently employed for wages or self-employed), county urbanization, and state Medicaid expansion in 2014, and to provide estimates specifically reflecting the association between occupation and being uninsured, adjusted prevalences were estimated using logistic regression. The initial model included occupational group, year, the interaction between occupational group and year, confounders, and the two-way interaction term between each confounder and year. County urbanization and each confounder interaction term except for age by year and income by year were dropped from the final model because they were not statistically significant.

Among the 17 states, the survey response rates ranged from 31.1% to 59.2% in 2013^††^ and 33.0% to 57.6% in 2014.^§§^ In 2013 and 2014, the subpopulation of interest comprised 138,407 workers. Among these, 18,140 (13%) were excluded because occupation was missing or could not be coded, leaving 59,718 respondents in 2013 and 60,549 in 2014.

The overall prevalence of being uninsured among workers in 2013 (16.0%) declined 21% (p<0.001) (3.3 percentage points [p<0.001]) to 12.7% in 2014 (Table 1). The prevalence of being uninsured declined in all demographic groups in 2014; both the percentage point difference and the percentage change were statistically significant for all groups except persons aged 25–34 years, persons who took the survey in Spanish, those with household incomes ≥$50,000, and those who resided in urban and rural counties. The decline was statistically significant among workers who lived in the most populous counties, metropolitan (Table 1). The prevalence of being uninsured exceeded 20% in 2014 (well above the average of 12.7%) among workers who took the survey in Spanish, who had less than a high school education, who had an annual household income <$35,000, were of Hispanic ethnicity, self-employed, and never married.

**TABLE 1:** Prevalence* of not having health insurance among currently employed workers, by selected characteristics and year — Behavioral Risk Factor Surveillance System, 17 states, 2013–2014

Characteristic	2013		2014		2013 to 2014	
	No. in sample	Uninsured % (95% CI)	No. in sample	Uninsured % (95% CI)	Percentage point difference % (95% CI)	Percent change^†^ %
**Age group (yrs)**						
18–24	3,566	26.6 (23.7–29.8)	3,774	18.6 (16.0–21.7)	-8.0 (-12.2 to -3.8)	-30^§^
25–34	9,276	20.8 (19.1–22.6)	9,226	19.6 (17.7–21.7)	-1.2 (-3.9 to 1.5)	-6
35–44	12,395	16.6 (15.0–18.3)	12,297	12.8 (11.5–14.3)	-3.8 (-6.0 to -1.6)	-23^§^
45–54	17,256	11.5 (10.5–12.7)	17,057	8.1 (7.1–9.2)	-3.5 (-5.0 to -1.9)	-30^§^
55–64	17,225	9.4 (8.5–10.5)	18,195	6.8 (5.9–7.8)	-2.6 (-4.0 to -1.3)	-28^§^
**Sex**						
Men	27,835	18.7 (17.6–19.9)	28,766	15.5 (14.4–16.7)	-3.2 (-4.8 to -1.6)	-17^§^
Women	31,883	12.8 (11.9–13.6)	31,783	9.5 (8.7–10.3)	-3.3 (-4.5 to -2.1)	-26^§^
**Race/Ethnicity**						
White, non-Hispanic	48,198	11.1 (10.5–11.7)	48,557	8.6 (7.9–9.3)	-2.5 (-3.4 to -1.6)	-23^§^
Black, non-Hispanic	3,746	20.3 (17.9–22.9)	3,878	15.5 (13.7–17.5)	-4.8 (-8.0 to -1.6)	-24^§^
Other, non-Hispanic	3,166	17.6 (14.1–21.8)	3,237	12.4 (9.6–15.9)	-5.2 (-10.2 to -0.2)	-29^§^
Hispanic	4,026	38.3 (35.1–41.6)	4,176	33.3 (30.1–36.8)	-5.0 (−9.6 to -0.3)	-13^§^
**Survey language**						
English	58,591	13.7 (13.1–14.4)	59,433	10.4 (9.8–11.1)	-3.3 (-4.3 to -2.4)	-24^§^
Spanish	1,047	60.7 (54.9–66.1)	1,099	58.4 (52.8–63.8)	-2.3 (-10.2 to 5.6)	-4
Other	44	^¶^	2	^¶^	—	—
**Education**						
Less than high school	2,271	48.2 (44.0–52.5)	2,334	41.4 (37.3–45.7)	-6.8 (-12.7 to -0.8)	-14^§^
High school graduate	13,521	20.8 (19.4–22.3)	13,806	17.0 (15.4–18.7)	-3.8 (-6.0 to -1.6)	-18^§^
Some college or technical school	16,847	14.1 (13.0–15.3)	16,937	11.0 (9.9–12.2)	-3.1 (-4.7 to -1.5)	-22^§^
College graduate or more	27,023	5.5 (5.0–6.2)	27,399	3.4 (3.0–3.9)	-2.1 (-2.9 to -1.3)	-38^§^
**Annual household income**						
$0–$14,999	2,153	49.3 (44.5–54.1)	2,110	37.8 (32.7–43.1)	-11.5 (-18.6 to -4.4)	-23^§^
$15,000–$24,999	5,753	43.0 (40.1–46.0)	5,549	31.8 (29.0–34.7)	-11.2 (-15.3 to -7.1)	-26^§^
$25,000–$34,999	4,911	27.2 (24.5–29.9)	4,679	22.4 (19.3–25.8)	-4.8 (-9.0 to -0.6)	-18^§^
$35,000–$49,999	7,739	17.2 (15.0–19.7)	7,448	12.8 (11.1–14.7)	-4.4 (-7.3 to -1.4)	-25^§^
$50,000–$74,999	10,676	7.6 (6.4–8.9)	10,323	6.8 (5.4–8.6)	-0.7 (-2.8 to 1.3)	-10
≥$75,000	24,327	3.2 (2.7–3.8)	25,492	3.0 (2.4–3.9)	-0.2 (-1.1 to 0.7)	-5
**Marital status**						
Married	36,250	9.5 (8.7–10.4)	37,602	6.8 (6.2–7.6)	-2.7 (-3.8 to -1.6)	-28^§^
Divorced, widowed, or separated	10,385	22.8 (20.9–24.7)	9,832	17.8 (16.1–19.7)	-4.9 (-7.5 to -2.3)	-22^§^
Never married or a member of an unmarried couple	12,868	24.9 (23.3–26.6)	12,893	21.3 (19.5–23.1)	-3.6 (-6.1 to -1.2)	-15^§^
**Employment status**						
Employed for wages	50,776	13.8 (13.1–14.5)	51,382	11.2 (10.5–11.9)	-2.6 (-3.6 to -1.6)	-19^§^
Self-employed	8,942	29.5 (27.1–32.0)	9,167	22.4 (19.9–25.1)	-7.1 (-10.7 to -3.5)	-24^§^
Metropolitan/Urban/Rural county of residence**						
Metropolitan	41,803	15.6 (14.8–16.4)	42,527	12.2 (11.4–13.0)	-3.4 (-4.5 to -2.2)	-22^§^
Urban	14,360	18.0 (16.6–19.6)	14,403	15.9 (14.1–18.0)	-2.1 (-4.5 to 0.4)	-11
Rural	3,555	18.4 (15.8–21.3)	3,619	15.0 (12.2–18.3)	-3.4 (-7.5 to 0.7)	-18
**State Medicaid expansion in 2014**						
State did expand^††^	42,670	15.3 (14.5–16.1)	41,085	11.8 (11.0–12.6)	-3.5 (-4.6 to -2.3)	-23^§^
State did not expand^§§^	17,048	20.1 (18.8–21.4)	19,464	18.3 (17.3–19.4)	-1.7 (-3.4 to -0.1)	-9^§^
**Total**	**59,718**	**16.0 (15.3–16.7)**	**60,549**	**12.7 (12.0–13.4)**	**-3.3 (-4.3 to -2.4) ^§^**	**-21^§^**

**Abbreviation:** CI = confidence interval.

* Unadjusted, weighted estimates.

^†^ Percent change = [(prevalence in 2014 – prevalence in 2013)/prevalence in 2013] x 100.

^§^ p<0.05.

^¶^ Estimates have a relative standard error >50% and are not shown as they do not meet standards of reliability/precision.

** County of residence was classified as metropolitan (codes 1–3), urban (4–7), or rural (8–9), based on the U.S. Department of Agriculture’s 2013 Rural-Urban Continuum Codes. https://www.ers.usda.gov/data-products/rural-urban-continuum-codes.aspx.

^††^ Except as noted, expansion began January 1, 2014: Illinois, Maryland, Massachusetts, Michigan (April 1, 2014), Minnesota, New Hampshire (August 15, 2014), New Jersey, New Mexico, New York, North Dakota, Oregon, and Washington (n = 83,755; 69.6% of respondents).

^§§^ Louisiana, Mississippi, Montana, Nebraska, and Utah (n = 36,512; 30.4% of respondents).

In both 2013 and 2014, a lower percentage of workers were uninsured in the 12 states that expanded Medicaid eligibility than were in the five states that did not, and the prevalence of being uninsured declined more (23%) in states that expanded Medicaid than in those that did not (9%; p = 0.013), although the percentage point difference between the two groups of states was not statistically significant (Table 1).

In both 2013 and 2014, there were statistically significant differences among occupation groups in the unadjusted prevalence of being uninsured (p<0.001). In 2013, the unadjusted prevalence of being uninsured ranged from 3.6% among the architecture and engineering occupations to 37.9% among the farming, fishing, and forestry occupations. In 2014, the unadjusted prevalence of being uninsured ranged from a high of 37.0% (building and grounds cleaning and maintenance) to 2.7% (community and social services; and education, training, and library) (Table 2). More than 25% of the workers in four occupations (construction and extraction [29.1%]; farming, fishing, and forestry [34.6%]; food preparation and serving related [35.5%]; and building and grounds cleaning and maintenance [37.0%]) reported not having health insurance in 2014.

**TABLE 2:** Prevalence* of not having health insurance among currently employed workers aged 18 to 64 years, by occupational group^†^ and year, ranked from lowest to highest prevalence in 2014 — Behavioral Risk Factor Surveillance System, 17 states, 2013–2014

Occupational group	2013		2014		2013 to 2014	
	No. in sample	Uninsured % (95% CI)	No. in sample	Uninsured % (95% CI)	Percentage point difference % (95% CI)	Percent change^§^ %
Community and social services	1,426	6.6 (4.6–9.5)	1,391	2.7 (1.6–4.3)	-4.0 (-6.7 to -1.3)	-60^¶^
Education, training, and library	5,171	5.2 (3.9–6.8)	5,215	2.7 (1.8–4.0)	-2.5 (-4.3 to -0.7)	-48^¶^
Healthcare practitioners and technical	5,275	4.5 (3.5–5.8)	5,149	2.8 (2.1–3.7)	-1.7 (-3.1 to -0.3)	-38^¶^
Computer and mathematical	1,990	6.2 (3.3–11.5)**	2,211	3.3 (2.0–5.2)	-3.0 (-7.2 to 1.3)	-48
Life, physical, and social science	1,097	4.0 (2.2–7.2)**	1,139	3.4 (1.6–7.0)**	-0.6 (-4.1 to 2.8)	-16
Business and financial operations	2,778	3.7 (2.4–5.7)	2,530	3.9 (2.6–5.8)	0.2 (-2.1 to 2.4)	5
Architecture and engineering	1,726	3.6 (2.2–5.8)	1,735	4.4 (2.7–7.2)	0.9 (-1.9 to 3.6)	24
Protective service	1,169	9.9 (6.5–14.8)	1,185	5.4 (3.6–8.2)	-4.4 (-9.1 to 0.3)	-45^¶^
Legal	833	4.6 (2.8–7.7)	863	6.1 (2.4–14.6)**	1.5 (-4.5 to 7.5)	32
Management	6,914	9.7 (8.3–11.3)	7,450	6.8 (5.5–8.4)	-2.9 (-5.0 to -0.8)	-30^¶^
Office and administrative support	7,104	9.4 (7.8–11.3)	7,100	7.3 (5.9–9.1)	-2.1 (-4.5 to 0.3)	-22
Arts, design, entertainment, sports, and media	1,311	15.1 (11.7–19.3)	1,261	10.8 (7.2–16.0)	-4.3 (-10.1 to 1.4)	-29
Healthcare support	1,513	23.6 (18.6–29.4)	1,460	11.3 (8.7–14.6)	-12.2 (-18.4 to -6.1)	-52^¶^
Sales and related	5,198	19.3 (17.1–21.7)	5,286	12.6 (10.9–14.5)	-6.7 (-9.7 to -3.8)	-35^¶^
Installation, maintenance, and repair	1,826	18.5 (15.1–22.6)	1,909	16.2 (12.5–20.7)	-2.3 (-7.9 to 3.2)	-13
Production	2,661	18.1 (15.2–21.5)	2,530	16.4 (13.0–20.4)	-1.8 (-6.6 to 3.1)	-10
Personal care and service	1,910	23.8 (20.2–28.0)	1,974	16.7 (13.4–20.6)	-7.1 (-12.5 to -1.8)	-30^¶^
Transportation and material moving	2,604	26.7 (22.7–31.1)	2,656	21.7 (18.1–25.7)	-5.0 (-10.7 to 0.7)	-19
Construction and extraction	3,089	34.9 (31.1–38.9)	3,194	29.1 (25.3–33.3)	-5.8 (-11.4 to -0.2)	-17^¶^
Farming, fishing, and forestry	414	37.9 (28.6–48.1)	508	34.6 (23.7–47.5)	-3.3 (-18.9 to 12.3)	-9
Food preparation and serving related	1,789	37.4 (32.5–42.5)	1,804	35.5 (29.9–41.5)	-1.9 (-9.6 to 5.8)	-5
Building and grounds cleaning and maintenance	1,920	37.3 (32.2–42.7)	1,999	37.0 (31.6–42.8)	-0.3 (-8.0 to 7.4)	-1

**Abbreviation:** CI = confidence interval.

* Unadjusted, weighted estimates.

^†^ From the 2000 Standard Occupational Classification System. https://www.bls.gov/soc/.

^§^ Percent change = [(prevalence in 2014 – prevalence in 2013) / prevalence in 2013] x 100.

^¶^ p<0.05.

** Estimates have a relative standard error >30% and ≤50% and should be used with caution as they do not meet standards of reliability/precision.

There were also statistically significant differences in adjusted prevalence of being uninsured by occupation (p<0.001) in both 2013 and 2014. The 2014 adjusted prevalence of being uninsured ranged from 19.4% in the farming, fishing, and forestry occupations to 5.4% in the education, training, and library occupations. Half the occupational groups experienced significant decreases from 2013 to 2014 in the adjusted prevalence of being uninsured (Table 3). The same four occupational groups with the highest unadjusted prevalences of being uninsured in 2014 also had the highest prevalences in 2014 after adjustment for potential confounders (Table 3).

**TABLE 3:** Adjusted prevalence* of not having health insurance among currently employed workers aged 18 to 64 years by occupational group^†^ and year, ranked from lowest to highest prevalence in 2014 — Behavioral Risk Factor Surveillance System, 17 states, 2013–2014

Occupational group	2013		2014		2013 to 2014	
	No. in sample	Uninsured % (95% CI)	No. in sample	Uninsured % (95% CI)	Percentage point difference % (95% CI)	Percent change^§^ %
Education, training, and library	4,822	13.4 (10.6–16.7)	4,809	5.4 (4.1–7.0)	-8.0 (-11.3 to -4.7)	-60^¶^
Community and social services	1,358	11.3 (7.9–16.0)	1,295	5.9 (3.8–9.0)	-5.5 (-10.2 to -0.7)	-48^¶^
Protective service	1,100	11.6 (8.8–15.2)	1,107	6.4 (4.1–10.1)	-5.2 (-9.5 to -0.9)	-45^¶^
Computer and mathematical	1,846	14.8 (9.4–22.5)	2,030	6.7 (4.3–10.4)	-8.1 (-15.1 to -1.0)	-55^¶^
Healthcare practitioners and technical	4,924	12.2 (9.9–14.9)	4,760	6.9 (5.1–9.1)	-5.3 (-8.4 to -2.2)	-44^¶^
Life, physical, and social science	1,038	9.5 (5.9–14.9)	1,059	7.8 (4.3–13.7)	-1.6 (-7.9 to 4.6)	-17
Healthcare support	1,378	18.5 (14.3–23.5)	1,306	8.4 (6.0–11.5)	-10.1 (-15.3 to -4.8)	-55^¶^
Office and administrative support	6,471	11.7 (10.1–13.5)	6,427	8.6 (6.9–10.7)	-3.1 (-5.5 to -0.6)	-26^¶^
Business and financial operations	2,624	9.1 (6.7–12.1)	2,356	9.4 (6.8–13.0)	0.4 (-3.6 to 4.4)	4
Arts, design, entertainment, sports, and media	1,208	16.3 (12.8–20.4)	1,124	9.5 (6.4–13.8)	-6.8 (-12.0 to -1.6)	-42^¶^
Architecture and engineering	1,596	8.8 (4.5–16.4)**	1,604	9.9 (6.5–14.9)	1.2 (-5.9 to 8.2)	13
Personal care and service	1,700	15.6 (12.9–18.7)	1,761	10.3 (8.1–13.0)	-5.3 (-9.1 to -1.6)	-34^¶^
Management	6,453	15.1 (13.0–17.4)	6,848	10.7 (8.7–13.1)	-4.4 (-7.5 to -1.3)	-29^¶^
Sales and related	4,718	17.4 (15.4–19.6)	4,665	11.3 (9.8–13.1)	-6.1 (-8.7 to -3.5)	-35^¶^
Production	2,414	12.7 (10.9–14.8)	2,273	12.0 (9.6–14.7)	-0.7 (-3.9 to 2.4)	-6
Installation, maintenance, and repair	1,696	15.0 (12.6–17.9)	1,731	12.8 (9.5–17.1)	-2.2 (-6.8 to 2.4)	-15
Transportation and material moving	2,351	16.7 (14.1–19.6)	2,356	13.8 (11.1–17.2)	-2.8 (-6.9 to 1.3)	-17
Legal	778	13.7 (9.3–19.8)	800	14.9 (7.3–28.1)**	1.2 (-10.2 to 12.7)	9
Building and grounds cleaning and maintenance	1,690	16.9 (13.9–20.4)	1,749	16.7 (13.8–20.0)	-0.2 (-4.6 to 4.2)	-1
Construction and extraction	2,845	21.5 (19.1–24.2)	2,876	18.6 (15.9–21.6)	-2.9 (-6.6 to 0.7)	-14
Food preparation and serving related	1,534	19.7 (16.8–22.9)	1,511	19.3 (15.5–23.8)	-0.3 (-5.4 to 4.8)	-2
Farming, fishing, and forestry	375	15.8 (10.4–23.3)	429	19.4 (12.5–28.9)	3.6 (-6.7 to 13.9)	23

**Abbreviation:** CI = confidence interval.

* Weighted estimates adjusted by age group, sex, race/ethnicity, language in which the survey was administered (English, Spanish, other), education, annual household income, marital status, employment status (currently employed for wages or self-employed), and state Medicaid expansion.

^†^ From the 2000 Standard Occupational Classification System. https://www.bls.gov/soc/.

^§^ Percent change = [(prevalence in 2014 – prevalence in 2013) / prevalence in 2013] x 100.

^¶^ p<0.05.

** Estimates have a relative standard error >30% and ≤50% and should be used with caution as they do not meet standards of reliability/precision.

## Discussion

Among currently employed workers aged 18–64 years in 17 U.S. states, the overall percentage who did not have health insurance decreased significantly (21% decline) from 2013 to 2014. This finding is consistent with the decline in being uninsured among all U.S. adults from 20.4% in 2013 to 16.3% in 2014 (*8*). The 1-year changes were significant for some occupations. During both years, the prevalence among workers of not having health insurance varied by occupation, and this variation persisted after adjustment for factors known to be associated with insurance coverage. Among the occupations with the highest worker prevalences of being uninsured were farming, fishing, and forestry and construction and extraction, two occupations that are also among the most hazardous (*9*).

During the study period, the requirement to obtain qualifying health insurance began in January 2014, and included, among others, an exemption if the minimum annual premiums exceeded 8% of household income^¶¶^; hence, some respondents might not have been able to afford coverage. There was no employer mandate to provide health insurance to employees in 2014,***^,^^†††^ which might have affected some respondents’ ability to obtain coverage. Workers who took the survey in Spanish had a particularly high prevalence of being uninsured, even in 2014, possibly because they did not qualify for Medicaid, could not afford coverage, or did not have employers who provided health insurance.

The findings in this report are subject to at least seven limitations. First, because they are not addressed by BRFSS, this study does not account for certain factors which might have affected workers’ access to health insurance and which might have affected occupations differentially; including them would have narrowed the differences in adjusted prevalences by occupation within a year. These include the number of workers employed by the employer and whether the worker worked full- or part-time, had a temporary or permanent job, or was a contract worker. Second, 15.5% and 10.2% of currently employed, age-eligible workers had uncodable or missing occupation information in 2013 and 2014, respectively, and were excluded from the analyses. However, there was no significant difference in insurance status in either year between those with and without occupation information. Third, all BRFSS data are self-reported and could not be verified. Fourth, households without telephones are excluded from BRFSS, and the prevalence of being uninsured varies by household telephone status.^§§§^^,^^¶¶¶^ However, this should have little impact on the findings because only an estimated 2.3% of households do not have telephones.**** Fifth, because of the overall low survey response rates among the 17 states in 2013 and 2014, nonresponse bias is possible. Sixth, because only 17 states used the industry and occupation module in both years, the findings might not be nationally representative. Finally, causality for the changes observed from 2013 to 2014 is beyond the scope of this study.

Because some workplace conditions (*10*) and health outcomes (*4*,*5*) vary by industry or occupation, workers might rely on health insurance for treatment of work-related injuries or illnesses, and health insurance coverage can influence health status (*1*,*2*) as well as the ability to remain employed, identifying factors affecting differences in insurance rates by occupation might help to target interventions to reduce health disparities among U.S. workers. Given the changes in health insurance coverage from 2013 to 2014 and the wide variability in coverage by occupation, BRFSS data could be used to monitor changes in insurance among workers over time by occupation (such as the effect of changes in Medicaid policy on workers’ health care coverage) and to assess associations between health outcomes and differences in coverage among occupations.

**Summary**

**What is already known about this topic?**

Lack of health insurance has been associated with poorer health status and with difficulties accessing preventive health services and obtaining medical care, especially for chronic diseases.

**What is added by this report?**

During 2014, 12.7% of workers aged 18–64 years were uninsured (21% decline from 2013); declines occurred in all demographic groups. By occupational group, the 2014 prevalence of not having health insurance ranged from 37.0% (building and grounds cleaning and maintenance) to 2.7% (community and social services; and education, training, and library).

**What are the implications for public health practice?**

Identifying factors affecting differences in insurance rates by occupation might help to target interventions to reduce health disparities among U.S. workers.

## Footnotes

*
https://www.cdc.gov/brfss/ .

† Illinois, Louisiana, Maryland, Massachusetts, Michigan, Minnesota, Mississippi, Montana, Nebraska, New Hampshire, New Jersey, New Mexico, New York, North Dakota, Oregon, Utah, and Washington.

§ Health care coverage was elicited by the question, “Do you have any kind of health care coverage, including health insurance, prepaid plans such as HMOs, government plans such as Medicare, or Indian Health Service?” Possible responses were: yes, no, don’t know/not sure, refused.

¶ Occupation was elicited by the question, “What kind of work do you do—for example, registered nurse, janitor, cashier, auto mechanic?”

** Patient Protection and Affordability Act, Pub L. No. 111–148, 124 Stat. 271, (March 2010).

†† https://www.cdc.gov/brfss/annual_data/2013/pdf/2013_dqr.pdf.

§§ https://www.cdc.gov/brfss/annual_data/2014/pdf/2014_dqr.pdf.

¶¶ https://www.irs.gov/affordable-care-act/the-individual-shared-responsibility-payment-an-overview.

*** https://www.irs.gov/affordable-care-act/employers/employer-shared-responsibility-provisions.

††† 79 Fed. Reg. 8543 (February 12, 2014). https://www.federalregister.gov/documents/2014/02/12/2014-03082/shared-responsibility-for-employers-regarding-health-coverage.

§§§
https://www.cdc.gov/nchs/data/nhis/earlyrelease/wireless201312.pdf .

¶¶¶
https://www.cdc.gov/nchs/data/nhis/earlyrelease/wireless201412.pdf .

**** https://www.cdc.gov/brfss/annual_data/2014/pdf/overview_2014.pdf.
